# Composition of Volatiles, Phytochemical Analysis, Antioxidant and Anticancer Activity of *Euryops floribundus* Ne.Br. Leaves (Asteraceae)

**DOI:** 10.3390/molecules30234555

**Published:** 2025-11-26

**Authors:** Zoleka Mhinana, Buyisile Mayekiso, Siphamandla Lamula, Thando Bhanisa, Martha Wium, Juliano Paccez, Luiz Zerbini, Callistus Bvenura, Lisa Buwa-Komoreng

**Affiliations:** 1 Department of Biotechnology and Biological Sciences, Faculty of Science and Agriculture, University of Fort Hare, Private Bag X1314, Alice 5700, South Africa; zmhinana@ufh.ac.za (Z.M.); bmayekiso@ufh.ac.za (B.M.); 201927865@ufh.ac.za (T.B.); lbuwa@ufh.ac.za (L.B.-K.); 2 Infectious Diseases and Medicinal Plants Research Niche Area, Department of Biotechnology and Biological Sciences, Faculty of Science and Agriculture, University of Fort Hare, Private Bag X1314, Alice 5700, South Africa; 3 International Centre for Genetic Engineering and Biotechnology (ICGEB), Weirner & Beit Building, Anzio Rd, Observatory, Cape Town 7935, South Africa; mariet.wium@icgeb.org (M.W.); juliano.paccez@icgeb.org (J.P.); luiz.zerbini@icgeb.org (L.Z.); 4 Horticultural Sciences Department, Faculty of Applied Sciences, Cape Peninsula University of Technology, Symphony Way, Bellville, Cape Town 7535, South Africa; bvenurac@cput.ac.za

**Keywords:** Anticancer, *Euryops floribundus*, volatiles, antioxidants, FTIR, phytochemical analysis

## Abstract

*Euryops floribundus* has traditionally been recognized for its therapeutic benefits, although its pharmacological potential has not been fully explored. This study investigated the leaves of *E. floribundus* for volatile compounds, in vitro anticancer potential, antioxidant activity, and phytochemical composition using standard methods. Terpenoids, flavonoids, glycosides, tannins, saponins, and steroids were identified using qualitative screening. FTIR analysis verified that the functional groups corresponded to bioactive substances. Methanol, ethanol, and aqueous extracts showed dose-dependent 2,2-diphenyl-1-picrylhydrazyl (DPPH) radical scavenging activity. Nitric oxide scavenging activity ranged from 7.2% to 22.5% at 2500 μg/mL. GC-MS profiling of essential oils revealed monoterpenes as the primary constituents (60%), with sabinene (27.86%) and terpinen-4-ol (13.63%) as major chemicals. Significant antiproliferative effects were shown by ethanol and methanol extracts against DU-145, PC-3, and SK-UT-1 cancer cell lines with cell viability inhibition ranging from 82.6% to 85.6%, and IC_50_ values ranging from 1.7 ± 0.02 to 2.7 ± 0.03 µg/mL. These findings support the potential therapeutic uses of *E. floribundus* leaves and necessitate additional bioassay-guided research since they indicate that the leaves are rich in bioactive phytochemicals with antioxidant and anticancer activities.

## 1. Introduction

The pharmacological significance of therapeutic plants is derived from their bioactive phytochemical constituents, which have physiological effects on the human body. Alkaloids, essential oils, flavonoids, tannins, and terpenoids are among the most important phytochemical groups of organic substances that constitute the foundation for many contemporary synthetic medications that are still used today [1]. These metabolites have a variety of modes of action, including disrupting microbial membranes (e.g., carvacrol, thymol, eugenol), suppressing biofilm growth, inhibiting capsule synthesis, and reducing microbial toxin production [2]. Flavonoids and terpenoids have been recognized for their diverse biological and therapeutic activities, such as antioxidant, antibacterial, anti-inflammatory, and anticancer effects [3]. They are essential for preventing infectious and degenerative diseases as well as reducing the negative effects of oxidative stress. Similarly, essential oils derived from aromatic plants are valued for their antimicrobial, antifungal, antioxidant, and anticarcinogenic properties [3,4], and are frequently used in the food sector as natural flavorings [5]. The extraction of these secondary metabolites from various plant organs such as leaves, peels, flowers, and roots has shown promising medicinal properties. However, the growth of antibiotic-resistant bacteria and cancer cell’s increasing resistance to conventional treatment highlights the critical need for innovative plant-derived bioactive compounds. Indigenous South African communities recognize the therapeutic significance of various plant families, including the Asteraceae, Lamiaceae, Solanaceae, Geraniaceae, and Cannabaceae.

The Asteraceae family, also known as the sunflower family, is one of the largest groups of flowering plants, with over 1600 genera and over 25,000 species worldwide [6]. This family includes economically and pharmacologically significant plants such as sunflower, chicory, chamomile, and dandelion [7]. Several Asteraceae species have been shown to possess chemically important natural compounds with great therapeutic potential, including anti-inflammatory, antibacterial, antioxidant, hepatoprotective, and anticancer effects [5].

*Euryops floribundus* (Kamdeboo resin bush) is a perennial shrub in the Asteraceae family that grows in the rocky terrains of the Eastern Cape Province in South Africa. It is distinguished by its narrow, linear leaves and high resin content, and it has historically been appreciated for its medicinal and preservation properties [8]. Several South African communities have long utilized *E. floribundus* to treat wound infections, fever, stomach problems, and respiratory issues. According to historical records, both the Khoi people and early European settlers utilized the plant’s resin to replace gum and preserve leather items. In parts of KwaZulu-Natal and Limpopo, decoctions of the leaves are used for inflammation, skin infections, and general detoxification. Contemporary ethnobotanical surveys report its continued use for treating diarrhea, menstrual discomfort, and microbial infections, highlighting its relevance in primary healthcare practices (unpublished survey). Although deemed invasive in some regions due to its allelopathic potential and influence on grazing fields [9,10], *E. floribundus* remains an ecologically and pharmacologically interesting species [10]. Nonetheless, many species of this family are important as ornamental, medicinal and aromatic plants. Additionally, aromatic plants produce essential oils used in folk and modern medicine [11,12,13]. Given the Asteraceae family’s vast therapeutic value and the lack of scientific data on *E. floribundus*, the aim of this study was to assess the phytochemical composition, antioxidant activity, and anticancer potential of *E. floribundus* leaf extracts. The findings are intended to shed light on the pharmacological foundation for the plant’s traditional applications and identify prospective chemicals for future medicinal research.

## 2. Results

### 2.1. Phytochemical Analysis

Table 1 shows a qualitative analysis of *E. floribundus* leaf extracts that revealed the existence of several bioactive secondary metabolites. Terpenoids, flavonoids, glycosides, tannins, saponins, and steroids were found in all extracts, but alkaloids and anthraquinones were lacking. Water and methanol extracts had the richest phytochemical profiles as compared to ethanol and chloroform extracts, indicating that polar molecules are more soluble in these solvents.

### 2.2. Fourier-Transform Infrared Spectroscopy (FTIR)

#### 2.2.1. Aqueous Extract

**Figure 1:**
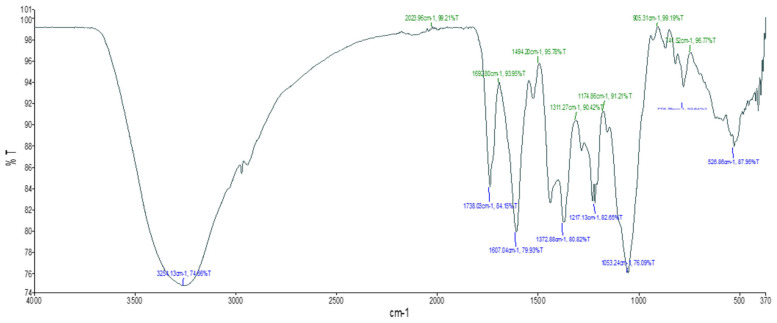
FTIR shows the functional group synthesized by the *E. floribundus* leaf aqueous extract.

The FTIR study of *E. floribundus* aqueous leaf extract revealed the presence of alcohol, carboxylic acids, and halogenated organic compounds. The spectra revealed the characteristic O-H stretching of intermolecularly bound alcohol at 3254 cm^−1^. The absorption at 1217 cm^−1^ suggested C-O stretching of carboxylic acids, while 1053 cm^−1^ indicated primary alcohols (Figure 1, Table 2).

#### 2.2.2. Dry Powder

**Figure 2:**
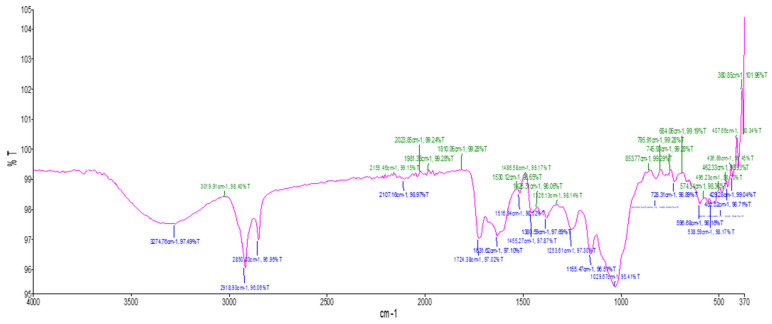
FTIR spectra of functional groups synthesized by *E. floribundus* dry powder.

The FTIR spectra of dried *E. floribundus* leaf powder showed the presence of alcohols, alkyl halides, carboxylic acids, saturated aliphatic compounds, and ketones (Figure 2, Table 3). The key absorptions were O-H stretching at 3274.76 cm^−1^ (carboxylic acids), asymmetrical and symmetrical -CH stretching at 2918.93 cm^−1^ and 2850.80 cm^−1^, and C=O ester stretching at 1724.38 cm^−1^ and 1631.62 cm^−1^. The fingerprint region showed aromatic C=C stretching, O-H bending, and C-N stretching. Peaks ranging from 429.28 cm^−1^ to 728.31 cm^−1^ indicate the presence of alkyl halides.

### 2.3. Essential Oil Composition

Hydrodistillation of aerial parts of *E. floribundus* yielded a colorless essential oil at 0.8% (*w*/*w*). GC-MS analysis identified 24 compounds representing 98.55% of the total essential oil. The dominant constituents were sabinene (27.86%), terpinene-4-ol (13.63%), α-pinene (9.53%), palmitic acid (8.64%), trans-β-ocimene (8.06%), γ-terpinene (5.02%), and α-terpinene (3.94%) (Table 4).

### 2.4. Antioxidant Activity

**Figure 3:**
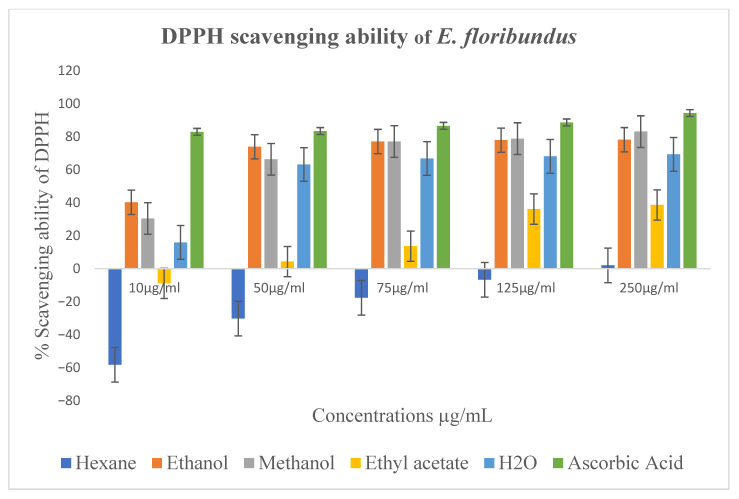
DPPH radical scavenging activity (%) of hexane, ethanol, methanol, ethyl acetate, and aqueous leaf extracts of *E. floribundus*. Negative scavenging values indicate an increase in absorbance relative to the control. Error bars represent standard deviation of three independent experiments performed in triplicate.

The 2,2-diphenyl-1-picrylhydrazyl (DPPH) and NO radical scavenging assays were used to assess the antioxidant properties of different extracts. Hexane and ethyl acetate extracts demonstrated minimal DPPH scavenging activity, while the methanol extract demonstrated significant activity at 250 µg/mL (Figure 3). For ethanol, methanol, ethyl acetate, and aqueous extracts, the IC_50_ values were 0.56 ± 3.2, 1.54 ± 1.9, 2.26 ± 4.1, and 2.41 ± 3.4 µg/mL, respectively (Table 5). Negative scavenging values indicate an increase in absorbance relative to the control.

Nitric oxide radicals by the aqueous, ethanol, and methanol extracts, as well as by ascorbic acid. At the highest tested concentration (2500 µg/mL), methanol, aqueous, and ethanol extracts exhibited scavenging activities of 7.2%, 19.9%, and 22.5%, respectively, while ascorbic acid showed 36.4% activity at the same concentration. The calculated IC_50_ values were 514.5 ± 13.8 µg/mL for the methanol extract, 316.1 ± 10.9 µg/mL for the aqueous extract, and 386.9 ± 12.0 µg/mL for the ethanol extract, whereas ascorbic acid demonstrated an IC_50_ of 431.4 ± 12.6 µg/mL (Table 5). All the extracts demonstrated relatively weak NO scavenging capacity, the aqueous extract exhibited the highest activity among the plant samples (Table 5).

### 2.5. In Vitro Anticancer Activity

**Figure 4:**
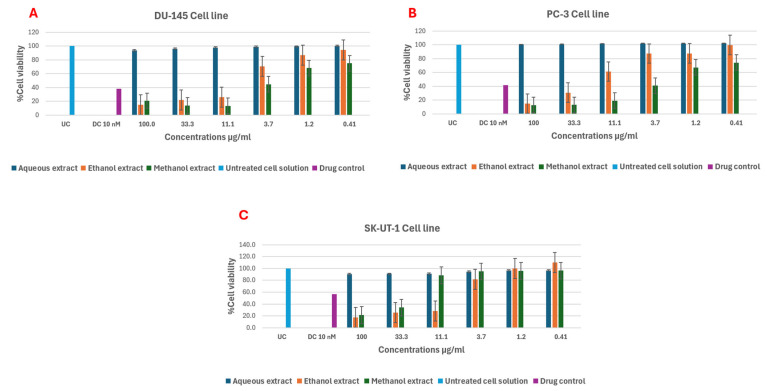
Inhibitory activity of *E. floribundus* leaf extracts against (**A**) DU-145, (**B**) PC-3, and (**C**) SK-UT-1 cell lines, expressed as % relative to untreated control (UC) and drug control (DC; docetaxel, Taxotere). Error bars represent standard deviation of three independent experiments performed in triplicate.

Aqueous, ethanol, and methanol extracts of *E. floribundus* were tested against prostate cancer cell lines (DU-145 and PC-3) and uterine leiomyosarcoma (SK-UT-1) using the MTT assay. Ethanol and methanol extracts significantly reduced cell viability in a dose-dependent manner, whereas aqueous extract showed minimal effect (Figure 4, Table 6). The ethanol extract exhibited IC_50_ values of 2.1 ± 0.02 µg/mL (DU-145), 2.7 ± 0.03 µg/mL (PC-3), and 1.7 ± 0.02 µg/mL (SK-UT-1), with cell viability inhibition ranging from 82.6% to 85.3%. Methanol extract demonstrated significant cell viability inhibition activity against DU-145 and PC-3 cell line at 3.7 to 33.3 μg/mL (Figure 4A,B). The aqueous extract showed little to no response against DU-145, PC-3 and SK-UT-1 cell line at 0.41 to 100 μg/mL (Figure 4A–C).

## 3. Discussion

### 3.1. Phytochemical Analysis

The qualitative phytochemical examination of *E. floribundus* indicated the presence of various secondary metabolites, including tannins, flavonoids, steroids, terpenoids, cardiac glycosides, and saponins, which were most prevalent in the water extract. These phytochemical pharmacological properties, such as antibacterial, antioxidant, anti-inflammatory, and antiviral activity, have been extensively studied [6,14]. The presence of these phytochemicals serves as a chemical basis for *E. floribundus* historic therapeutic usage. Importantly, solvent polarity affected the extraction efficiency of specific phytochemicals. Polar solvents (water, methanol, and ethanol) were more effective in extracting flavonoids and phenolic compounds, whereas moderately polar to non-polar solvents (ethyl acetate and hexane) favored the extraction of terpenoids and other less polar constituents. This differential extraction most likely accounts for the variation in bioactivity seen among the extracts.

### 3.2. Fourier Transform Infrared (FTIR) Spectroscopy Analysis

The presence of important functional groups associated with bioactive secondary metabolites was confirmed by FTIR analysis. Both aqueous and dry powder extracts showed peaks corresponding to O–H, C=O, C–O, C–Cl, and C–I, which indicated the presence of alcohols, carboxylic acids, phenols, ketones, and alkyl halides (Figure 1 and Figure 2, Table 2 and Table 3). Usually, these functional groups are associated with antibacterial and antioxidant properties. For instance, carbonyl groups in ketones and carboxylic acids can aid in redox processes, while hydroxy groups in phenols and alcohols are known to donate hydrogen atoms to neutralize free radicals [14]. Despite being poorly researched, alkyl halides may have an impact on antibacterial activity by compromising the integrity of microbial membranes.

### 3.3. Gas Chromatograph Mass Spectroscopy of Volatiles

The peppery-smelling volatile constituents indicated in Table 4 are most likely responsible for the strong, spicy resinous odor found in the colorless essential oil produced from *E. floribundus*. The GC-MS study of the aerial parts revealed a mixture of twenty-four compounds accounting for 98.55% of the total essential oil, indicating a chemically diverse volatile profile. The dominant compounds were sabinene (27.86%), terpinene-4-ol (13.63%), and α-pinene (9.53%). Other compounds like γ-terpinene, α-terpinene, and palmitic acid were found in lower amounts. The predominance of monoterpenes and oxygenated terpenoids aligns with the typical phytochemical profile of the Asteraceae family and likely contributes to the plant’s defensive and pharmacological properties. Notably, α-pinene and terpinene-4-ol are reported to induce cytotoxicity through ROS generation, mitochondrial disruption, and membrane destabilization, suggesting a mechanistic link to the antioxidant and antiproliferative activities observed in ethanol and methanol extracts. Trace levels of germacrene D, known for anti-inflammatory and anticancer activity, may further contribute to the overall bioactivity profile [15]. In line with the reported DPPH and NO radical scavenging results, flavonoids and phenolic chemicals found in the leaf extract further enhance these volatiles by aiding redox control and free radical scavenging. Consequently, the combined effects of volatile and non-volatile phytochemicals operating through a diverse range of mechanisms, such as the neutralization of reactive oxygen species (ROS) and the modification of cellular redox equilibrium, are most likely the source of *E. floribundus*’s biological activity [16]. It should be mentioned, nonetheless, that GC-MS profiling only yields qualitative and semi-quantitative data based on relative peak areas, and thus cannot determine absolute compound concentrations or definitive contributions to biological activity. To ascertain the pharmacological significance of these chemicals in intricate biological systems and to validate their mechanistic role, Future research should integrate quantitative analyses using authentic standards, molecular docking, and purified-compound assays to validate the pharmacological significance of key volatiles [17].

### 3.4. In Vitro Antioxidant Activity

Hexane and ethyl acetate extracts were found to be inactive, whereas ethanol and methanol extracts demonstrated the best antioxidant activity, followed by the aqueous extract, according to the DPPH and NO radical scavenging assays (Figure 3, Table 5). This enhanced activity is attributable to the higher solubility of polar phytochemicals, such as flavonoids, phenolic acids, and oxygenated terpenoids in alcoholic solvents. These compounds are well documented for their ability to neutralize free radicals through electron transfer, hydrogen atom donation, and redox-modulating mechanisms, which explains the superior antioxidant potential of the ethanol and methanol extracts [18]. The varying extraction efficiency for these bioactive components is reflected in the solvent-dependent variation in antioxidant activity. These results suggest that *E. floribundus* leaves are a promising natural antioxidant source with prospective uses in food preservation, nutraceutical development, and herbal therapeutics. Negative scavenging values indicate an increase in absorbance relative to the control, which suggests that the hexane extract does not scavenge DPPH radicals and may instead interfere with the assay matrix. Nonetheless, future research should include quantitative phytochemical analysis using calibration curves with authentic standards and bioactivity-guided fractionation to identify the specific compounds responsible for the observed effects.

### 3.5. In Vitro Anticancer Activity

Ethanol and methanol extracts had strong antiproliferative effects on DU-145, PC-3, and SK-UT-1 cell lines, whereas the aqueous extracts had the least activity (Figure 4, Table 6). These variations are consistent with the ability of alcohol-based solvents to extract a broader array of cytotoxic phytochemicals, including terpenoids, flavonoids, and phenolic acids. A plausible biochemical basis for the observed anticancer effects is provided by several of the major volatile constituents found in the extracts, such as α-pinene and terpinene-4-ol, which are known to induce cell-cycle arrest, reactive oxygen species (ROS) generation, and mitochondrial-dependent apoptosis [16,17,19,20,21]. Potential synergistic interactions among multiple terpenoids may further enhance the antiproliferative activity, although this requires experimental verification. Despite these promising results, interpretation should remain cautious because cytotoxicity assessment relied solely on the MTT assay, which measures metabolic activity but cannot distinguish between apoptosis, necrosis, autophagy, or growth arrest. Future studies should use complementary assays such as Annexin V/PI flow cytometry, caspase-3/7 activation assays, mitochondrial membrane potential (ΔΨm) analysis, and gene or protein expression profiling of apoptosis-related markers to fully elucidate the underlying mechanisms of cell death. Future research should also incorporate pure phytochemical bioassays, molecular docking, and fractionation-based techniques to identify the principal compounds responsible for the observed antiproliferative activities, such as sabinene, α-pinene, or terpinene-4-ol.

### 3.6. Limitations and Future Directions

The in vitro design of the study and the absence of mechanistic validation of anticancer efficacy are its limitations. The positive control reduced cell viability to approximately 50%, indicating sub-maximal cytotoxicity. Using a higher concentration capable of reducing viability below 5% would better demonstrate maximal susceptibility; this is noted as another limitation of the current study. Furthermore, although bioactive compounds were identified by GC–MS and FTIR assays, it is yet unknown how each of these compounds contributed to the reported antioxidant and anticancer activities. To confirm and build on the results reported here, future studies should employ mechanistic tests, in vivo investigations, and extract fractionation.

## 4. Materials and Methods

### 4.1. Collection of Plant Material

*Euryops floribundus* aerial parts were taken at Nico Malan Pass in Eastern Cape Province, South Africa (32°29′0.20″ S, 26°50′0.13″ E), located near Seymour village. A voucher specimen (Voucher No. BUW031SMHI01) was prepared and deposited at the Giffen Herbarium, University of Fort Hare, South Africa. The University of Fort Hare Research Ethics Committee approved the plant collection and laboratory analysis (Reference No. BUW031SMHI01).

### 4.2. Preparation of the Extracts

The plant material was air-dried at room temperature and then ground into a fine powder with a mechanical grinder. Approximately 30 g of dried leaf powder was extracted in 300 mL of each solvent (hexane, ethanol, methanol, ethyl acetate, chloroform, and distilled water) using a Labcon platform shaker (Laboratory Consumables, Durban, South Africa) at room temperature for 24 h. The mixes were filtered using Whatman No. 1 filter paper. Organic extracts were concentrated under reduced pressure at 45 °C using a rotary evaporator (Cole Parmer SB 1100, Shanghai, China), whereas the filtrate from the water extract was evaporated to dryness using a freeze–dryer (Genevac LTD, BTP-3ES00X, IP Swich, Ipswich, UK). All the crude extracts were stored at −20 °C until use.

Stock solutions for anticancer assays were prepared by dissolving 0.04 g of crude ethanol, methanol, and hexane extracts in 2 mL dimethyl sulfoxide (DMSO), while water extracts were dissolved in 2 mL distilled water. The extracts were vortexed and sequentially filtered through 0.45 μm and 0.22 μm sterile filters, wrapped in foil, and stored at −20 °C until use.

### 4.3. Extraction of Essential Oil

Essential oil was obtained from 1 kg of fresh leaves via hydro-distillation using a Clevenger-type apparatus (Essential Oil Determination Apparatus (Clevenger Apparatus), SmartLabs, Boksburg, South Africa) for approximately 2 h in 1 L of distilled water. The oil was collected from the graduated arm of the Clevenger apparatus, dried over anhydrous sodium sulfate, and stored at 4 °C in sealed amber vials until GC–MS (AGILENT TY-73534 7200 Accurate-Mass Q-TOF GC/MS Mass Spectrometer, Urbana, IL, USA) analysis. The yield (%) of essential oil was calculated as follows:

$$\mathrm{Oil}\;\mathrm{yield}\;(\%)=\frac{\mathrm{Volume}\;\mathrm{of}\;\mathrm{oil}\;\mathrm{obtained}}{\mathrm{Mass}\;\mathrm{of}\;\mathrm{starting}\;\mathrm{material}}\times 100$$

### 4.4. Phytochemical Screening

The phytochemicals of *E. floribundus* were determined by adopting the standard methods as described by Harborne [22], Trease and Evans [23], Sofowora [24] and Edeoga [1]. Tests for alkaloids, flavonoids, terpenoids, saponins, anthraquinones, cardiac glycosides, and tannins were performed. The presence of phytochemicals was confirmed by color changes or precipitate formation upon the addition of appropriate reagents.

### 4.5. Fourier Transform Infrared Spectroscopy Analysis

The FTIR analysis was carried out to identify functional groups in the *E. floribundus* extracts. Ten mg of dried extract powder was mixed with 100 mg KBr and compressed into translucent pellets. Spectra were recorded using an FTIR spectrometer (PerkinElmer, Waltham, MA, USA) in the range of 400–4000 cm^−1^ with a resolution of 4 cm^−1^.

### 4.6. Gas Chromatograph Mass Spectroscopy of Volatiles

A Hewlett Packard (HP) 6890 GC system (LabWare, Inc, New Castle County, DE, USA) coupled with a 5973 Series Mass Selective Detector (MSD) (Agilent, Santa Clara, CA, USA) was used to analyze volatile constitutes. Helium was used as the carrier gas when injecting the samples in splitless mode. The oven temperature was programmed from 60 °C with an increment of 3 °C/min after a 3 min hold, while the injector temperature was kept at 220 °C. A capillary column (30 m × 0.25 mm, 0.25 μm film thickness) was used to accomplish separation. By contrasting the mass spectra and retention durations of the compounds with those of real standards and NIST library data, the compounds were identified. A HP 5973 Series Mass Selective Detector (MSD) recorded the mass spectra. The yield of the essential oil was calculated using the following formula:

Percentage yield of oil (%) = Mass of starting plant material (g)/Volume of oil obtained (mL) × 100

### 4.7. Antioxidant Assay

#### 4.7.1. DPPH Radical Scavenging Assay

The antioxidant activity of each solvent extract was determined using the DPPH (2,2-diphenyl-1-picrylhydrazyl) radical scavenging method as described by Madikizela and McGaw [25]. A 2.5 mL aliquot of 2 mM DPPH methanolic solution was mixed with varying concentrations of each extract (5–250 µg/mL). Ascorbic acid (1.76 g/100 mL methanol) served as a positive control. After 30 min of incubation in the dark at room temperature, absorbance was measured at 517 nm. Antioxidant activity was calculated as follows:

% DPPH scavenging activity = (Absorbance of sample − Absorbance of blank)/(Absorbance of control − Absorbance of Blank) × 100

where Absorbance of control is the absorbance of the DPPH radical + methanol; Absorbance of sample is the absorbance of DPPH radical + sample extract/standard.

#### 4.7.2. Nitric Oxide (NO) Scavenging Activity

The Nitric Oxide (NO) scavenging activity of the plant extracts were determined by the method outlined by Wintola and Afolayan [18]. A reaction combination of 2 mL 10 mM sodium nitroprusside in phosphate-buffered saline (pH 7.4) was coupled with 0.5 mL of extract or standard antioxidants (butylated hydroxytoluene [BHT] and gallic acid) at varied concentrations (50–500 µg/mL). After 2.5 h of incubation at 25 degrees Celsius, 0.1 mL of the reaction mixture was treated with 0.1 mL of sulfanilic acid reagent (0.33% in 20% acetic acid) and incubated for 5 min. Then, 1 mL of 0.1% naphthylenediamine dichloride was added and incubated for 30 min. The absorbance was measured at 540 nm, and the NO scavenging activity was determined using the same formula as above.

NO radical scavenging activity (%) = (Absorbance of sample − Absorbance of blank)/(Absorbance of control − Absorbance of Blank) × 100

where Absorbance of control is the absorbance of NO radicals + methanol and Absorbance of sample is the absorbance of NO radicals + extract or standard.

### 4.8. Anticancer Activity

Human prostate carcinoma (DU-145 and PC-3), and uterine leiomyosarcoma (SK-UT-1) cell line were obtained from the American Type Culture Collection (ATCC). Cell lines were maintained in Dulbecco’s Modified Eagles Medium (DMEM) containing 10% fetal bovine serum (FBS), 1 mM L-glutamine, 100 units/mL penicillin, and 100 μg/mL streptomycin and kept at 37 °C in a humidified 5% CO_2_ incubator (Thermos Fisher Scientific, Rockville, MD, USA). The visualization of DU-145, PC-3, and SK-UT-1 cells was counted according to the trypan blue staining method [26].

The plate was divided into three sections: background control (no cells, no treatment), cells, with no treatment (as in the 100% growth), cells in the same percentage of DMSO as the highest concentration of plant extract and cells treated with *E. floribundus* leaf extracts at different concentrations. The treatment was performed in triplicate.

The anticancer activity of the plant extracts was tested in vitro on DU-145, PC-3, and SK-UT-1 cell lines using a modified MTT (3-(4, 5-dimethylthiazol-2-yl)-2, 5-diphenyltetrazolium bromide) tetrazolium reduction assay as described by Mosmann [27]. Briefly, 6 × 10^3^ cells/well in 100 μL complete media were seeded into a 96-well cell culture plate and allowed to attach to the plate for 24 h. 100 μg/mL of the diluted plant extract was added to the 96-well culture plate and serial dilution was performed, with untreated cell media as a control. The 96-well cell culture plates were then incubated at 37 °C in a humidified 5% CO_2_ for 72 h. Following incubation, 10 μL MTT (2.5 mg/mL) was added to each well and incubated for another 3–4 h and after the experiment was stopped by adding a sodium dodecyl sulfate 10% in 0.1 N HCl solution to solubilize the formed formazan and left overnight. Optical density in the wells was read in a microplate reader (Thermo Multiskan Go, Waltham, MA, USA) at a wavelength of 595 nm [27]. The absorbance values obtained from the control (untreated cells) wells were averaged, and this value was considered as 100% cell viability.

Cell viability was calculated as follows:

$$\mathrm{Percentage}\mathrm{cell}\mathrm{viability}=\frac{\mathrm{Absorbance}\;\mathrm{of}\;\mathrm{sample}}{\mathrm{Absorbance}\;\mathrm{of}\;\mathrm{control}}\times 100\%$$

### 4.9. Statistical Analysis

Microsoft Excel 2013 Window was used for all statistical analyses and plotting of the graphs. Data are expressed as mean ± standard deviation (SD). Statistical significance was determined using Student’s *t*-test with *p* < 0.05 and *p* < 0.01 considered significant. All experiments were performed in triplicate.

## 5. Conclusions

This study is the first to give a comprehensive phytochemical and bioactivity profile of *Erythrina floribundus* leaf extracts and essential oils gathered in South Africa in the Eastern Cape Province. Gas Chromatograph-Mass Spectroscopy (GC-MS) revealed the presence of 24 volatile compounds, accounting for 98.55% of the total oil composition, dominated by sesquiterpenes and monoterpenes such as germacrene D, α-pinene, α-terpineol, and terpinene-4-ol. These compounds are known to have antibacterial, antioxidant, and anticancer activities, implying a potential involvement in the plant’s defense system and pharmaceutical applications. The antioxidant assays demonstrated significant free radical scavenging activities across all solvent extracts, notably in the methanol and ethanol fractions, indicating a high concentration of phenolic and flavonoid compounds. Furthermore, in vitro anticancer testing against human prostate (DU-145, PC-3) and uterine leiomyosarcoma (SK-UT-1) cancer cell lines demonstrated a dose-dependent cytotoxicity, indicating *E. floribundus* as a viable source of bioactive compounds with therapeutic potential. Overall, the findings support the traditional usage of *E. floribundus* and demonstrate its ethnopharmacological value. Using molecular and in vivo models, future studies should concentrate on identifying and describing the specific bioactive compounds that are responsible for the activities that have been seen.

## Figures and Tables

**Table 1:** Results of qualitative phytochemical screening of leaf extracts of *E. floribundus.*

Phytochemical	Aqueous	Methanol	Ethanol	Chloroform
Terpenoids	+	+	+	+
Flavonoids	+	+	+	+
Glycosides	+	+	+	+
Tannins	+	+	+	+
Saponins	+	+	+	+
Steroids	+	+	+	+
Alkaloids	−	−	−	−
Anthraquinones	−	−	−	−

(+)—indicates the presence; (−)—the absence of the phytochemical tested.

**Table 2:** FTIR shows the functional group synthesized by the *E. floribundus* leaf aqueous extract.

SI. No.	Absorption Peak (cm^−1^) (Test Sample)	Functional Groups
1	526.86	C-I, C-Cl
2	1053.24	C-O stretching
3	1217.13	C-O stretching
4	1372.88	O-H bend, Alcoholic group
5	1607.04	C=O stretch
6	1738.03	C=O stretching
7	3254.13	O-H stretch, Carboxylic group

**Table 3:** FTIR shows the functional group synthesized by the *E. floribundus* dry powder.

SI. No.	Absorption Peak (cm^−1^)	Functional Groups
1	429.28	C-Halogen
2	455.52	C-Halogen
3	538.59	C-I, C-Cl
4	596.68	C-I, C-Cl
5	728.31	C-Cl
6	1029.67	PO3 stretching
7	1155.47	C-N stretch
8	1253.61	C-N stretch
9	1380.59	O-H bend, alcoholic group
10	1455.27	C=C aromatic ring stretching
11	1516.94	C=C stretch
12	1631.62	C=O stretching
13	1724.38	C=O stretch
14	2107.16	Carbon-Carbon triple bond
15	2850.80	Symmetrical stretching of -CH_2_(CH_2_) vibration
16	2918.93	Asymmetric stretching of CH(CH_2_) vibration
17	3274.76	O-H stretch, Carboxylic group

**Table 4:** Essential oil composition of *E. floribundus.*

Retention Time	Constituent	% Total
10.6464	α-Thujene	1.01
10.8745	α-Pinene	9.53
12.3700	sabinene	27.86
12.4613	β-Pinene	1.61
13.0473	Myrcene	1.39
13.9426	α-Terpinene	3.97
14.7088	trans-β-Ocimene	8.06
15.0724	cis-Ocimene	1.17
15.4215	γ-Terpinene	5.02
16.4253	Terpinolene	1.24
19.2891	Terpinene-4-ol	13.63
19.7266	α-Terpinene	0.76
27.7060	Germacrene D	0.64
28.6867	σ-Cadinene	0.67
31.8052	T-Muurolo	0.95
31.9416	α-Gurjunene	1.88
37.8521	Cyclooctane,1,4-dimethyl-trans-	1.73
38.0106	Palmitic acid	8.64
38.3583	3-Ethyl-5-methyl-1-propyl-Cyclohexane	1.98
38.6926	2,3,4-trimethyl-4,5-methylenetetradecane	1.98
39.0258	cis, trans-1,2,3-trimethyl-cyclohexane	1.36
39.2887	1,1′-bicyclohexanyl, 2-propyl-, cis-	1.59
41.1914	3,7,11-trimethyldodeca-7(Z),10-diene	0.94

**Table 5:** Antioxidant activity of *E. floribundus* (IC_50_ in µg/mL). Ascorbic acid was used as the standard control.

Extracts	DPPH Assay (IC_50_ µg/mL)	NO Assay (IC_50_ in µg/mL)
Aqueous	2.41 ± 3.4	316.1 ± 10.9
Ethanol	0.56 ± 3.2	386.9 ± 12.0
Methanol	1.54 ± 1.9	514.5 ± 13.8
Ethyl acetate	2.26 ± 4.1	-
Ascorbic acid	-	431.4 ± 12.6

**Table 6:** IC_50_ of the plant extracts on cancer cells as determined by the MTT assay.

Cell Lines						
	DU-145	PC-3	SK-UT-1	DU-145	PC-3	SK-UT-1
Plant Extracts	IC_50_ μg/ml			% Inhibition at 100 µg/ml		
Aqueous	-	-	-	6.1	-	9.5
Ethanol	2.1 ± 0.02	2.7 ± 0.03	1.7 ± 0.02	84.1	85.3	82.6
methanol	3.2 ± 0.02	2.7 ± 0.51	2.2 ± 0.02	79.3	87.4	78.6

(-), Not found.

## Data Availability

All data generated or analyzed during this study are included in this published article.
